# Ocular Perfusion Pressure and Pulsatile Ocular Blood Flow in Normal and Systemic Hypertensive Patients

**DOI:** 10.5005/jp-journals-10008-1177

**Published:** 2015-01-15

**Authors:** Fabio N Kanadani, Carlos R Figueiredo, Rafaela Morais Miranda, Patricia LT Cunha, Tereza Cristina M Kanadani, Syril Dorairaj

**Affiliations:** Department of Ophthalmology, Eye’s Institute of Medical Science, University Hospital, Belo Horizonte, Minas Gerais, Brazil; Department of Ophthalmology, Santa Casa Hospital, Belo Horizonte, Minas Gerais, Brazil; Department of Ophthalmology, Medical Science University Belo Horizonte, Minas Gerais, Brazil; Department of Ophthalmology, Medical Science University Belo Horizonte, Minas Gerais, Brazil; Department of Ophthalmology, Eye’s Institute of Medical Science, University Hospital, Belo Horizonte, Minas Gerais, Brazil; Department of Ophthalmology, Mayo Clinic, Jacksonville Florida, USA

**Keywords:** Ocular perfusion pressure, Pulsatile ocular blood flow, Glaucoma.

## Abstract

**Purpose:** Glaucomatous neuropathy can be a consequence of insufficient blood supply, increase in intraocular pressure (IOP), or other risk factors that diminish the ocular blood flow. To determine the ocular perfusion pressure (OPP) in normal and systemic hypertensive patients.

**Materials and methods:** One hundred and twenty-one patients were enrolled in this prospective and comparative study and underwent a complete ophthalmologic examination including slit lamp examination, Goldmann applanation tonometry, stereoscopic fundus examination, and pulsatile ocular blood flow (POBF) measurements. The OPP was calculated as being the medium systemic arterial pressure (MAP) less the IOP. Only right eye values were considered for calculations using Student’s t-test.

**Results:** The mean age of the patients was 57.5 years (36-78), and 68.5% were women. There was a statistically significant difference in the OPP of the normal and systemic hypertensive patients (p < 0.05). The difference in the OPP between these groups varied between 8.84 and 17.9 mm Hg.

**Conclusion:** The results of this study suggest that although the systemic hypertensive patients have a higher OPP in comparison to normal patients, this increase does not mean that they also have a higher OBF (as measured by POBF tonograph). This may be caused by chronic changes in the vascular network and in the blood hemodynamics in patients with systemic hypertension.

**How to cite this article:** Kanadani FN, Figueiredo CR, Miranda RM, Cunha PLT, Kanadani TCM, Dorairaj S. Ocular Perfusion Pressure and Pulsatile Ocular Blood Flow in Normal and Systemic Hypertensive Patients. J Curr Glaucoma Pract 2015;9(1):16-19.

## INTRODUCTION

The two main theories for pathogenesis of the glaucomatous optic neuropathy (GON) are vascular and mechanical. Both have been defended by research groups over the last 150 years. In accordance with the mechanical theory, the increase in intraocular pressure (IOP) stretches the laminar beam and causes damage to the axons of the ganglion retinal cells, while the vascular theory considers the GON a consequence of insufficient blood supply, increase in IOP, or other risk factors that diminish the ocular blood flow (OBF). In congenital, acute angle close, secondary glaucoma, clearly the increase in IOP is enough to cause GON. However, innumerable findings of normal tension glaucoma (NTG) cannot satisfactorily be explained only by this theory. The fact is that the reduction of OBF frequently precedes the structural damage. It also occurs in other parts of the body of these glaucomatous patients, indicating that hemodynamic alterations can at least partially be primary. The biggest cause of this reduction is not atherosclerosis, but a vascular disequilibrium that leads to the reduction of the ocular perfusion pressure (OPP) and insufficient autoregulation.^1^

The reduction in OBF can have a decisive implication in the pathophysiology of many ocular illnesses like diabetic retinopathy, age-related retinopathy, pigmentary retinosis, myopia, and glaucoma. Abnormalities of the blood flow in glaucoma have been evaluated with diverse techniques, including angiofluoresceinography, colored Doppler, Doppler laser fluxometry, and pulsatile ocular blood flow (POBF) tonograph.^2^ Using the angiofluoresceinography, Hayreh et al^3^ demonstrated a zone of the short posterior ciliary’s arterioles that supply the posterior portion of the optic nerve. This area can be vulnerable to the ischemia when the OPP is reduced. The OPP is defined as median arterial pressure (MAP) less IOP (OPP = MAP - IOP).^4^ In agreement with the vascular theory, low systemic arterial pressure (AP) relative to IOP can lead to a low OPP, consequently, decreasing the perfusion of the optic nerve and leading to a glaucomatous loss of the visual field. On the other hand, systemic hypertension can increase the risk of damage to the small vessels of the optic disk.^5^ A reduction in MAP or an increase in IOP can diminish the ocular perfusion. The accurate mechanism of the regulation of IOP is unknown. If the autoregulation mechanisms are continuous, the sanguineous flow will remain steady with a substantial fall of the ocular perfusion.^4^

The pulse of the cardiovascular system is also expressed in the eye to each systolic-diastolic cycle. The blood flows in the vessels of the ocular globe during systole and continues to flow more slowly during diastole. This phenomenon, according to a relationship between volume and pressure, implies a maximum IOP during systole and a minimum IOP during diastole. In this way, this transitory changes of the IOP allow us to calculate changes in ocular volume. POBF tonograph is capable of catching minimum changes in the IOP pulse and to correlate them with the volume. However, it cannot determine the OBF of isolated parts of the intrinsic vascular net of the eye. Therefore, its principle is based on the total influx of blood in each cardiac systole.^6-9^ This method measures mainly the OBF in the choroids and anterior portion of the head of the optic nerve supplied by the posterior ciliary’s vessels, which provides 80 to 90% of the OBF.^2^

The aim of this study is to determine the OPP and OBF in normal and systemic hypertensive patients.

## MATERIALS AND METHODS

The study was performed in adherence to the guidelines of the declaration of Helsinki. The study protocols were approved by the Ethics Committee of Santa Casa of Belo Horizonte, and all patients have signed the informed consent.

One hundred and twenty-one patients were enrolled in this prospective and comparative study and underwent a complete ophthalmologic examination including history of previous ocular diseases, trauma or surgery, slit lamp examination, Goldmann applanation tonometry, stereoscopic fundus examination, and POBF measurements.

Two measurements of IOP and AP were taken with Goldmann applanation tonometer in a seated position, with a 2-minute interval between the measurements. The patients were divided into two groups: normal and systemic hypertensive. After 5 minutes in a seated position, OBF measurements were taken with the POBF tonograph.

The same examiner (FK) took all the measurements of the POBF in the patients after the topic instillation of cloridrate of proximetacain 5 mg. The POBF tonograph has one pneumotonometer with disposable tips that, in contact with the previously anesthetized cornea, measures the pulses of the IOP. During the examination, the POBF tonograph produces a sound that helps the examiner to capture five representative pulses of the IOP. If after 20 seconds, the equipment is not able to detect five complete pulses of the IOP, the examination is automatically interrupted. The POBF tonograph is capable of detecting initial tensional levels from 5 to 127 mm Hg. The data supplied after the detection of the five pulses of the IOP are: IOP, minimum IOP (in mm Hg), pulse amplitude, pulse volume [(PV) in micro liters], pulse rate [(PR) in beats per minute] and, POBF (micro liters per second).

### Inclusion Criteria

Normal patients were ≥ 35 years old with an absence of ocular or systemic diseases, no ocular surgeries, and no use of any systemic medications. Refraction could not be greater than + 6 diopters (D) spheric and 3 cylindric. They had to have a best corrected visual acuity of 20/40 or better, IOP ≤ 21 mm Hg and cup/disk ratio < 0.4, and an absence of asymmetry.

Systemic hypertensive patients were ≥ 35 years old with an absence of ocular pathologies and surgeries, and systolic systemic AP ≥ 140 mm Hg and diastolic ≥ 90 mm Hg or normal levels of systemic AP with use of antihypertensive medications. Refraction could not be greater than + 6D spheric and 3 cylindric. They had to have a best corrected visual acuity of 20/40 or better, IOP ≥ 21 mm Hg and cup/disk ratio < 0.4, and absence of asymmetry.

The OPP was calculated as being the MAP less the IOP. Only right eye values were considered for calculations using Student’s t-test.

### System Hemodynamics

The patients stayed in the sitting position for 10 minutes before all measurements. Systolic and diastolic blood pressures were measured on the upper arm by a calibrated tycos sphygmomanometer.

## RESULTS

**Graph 1:**
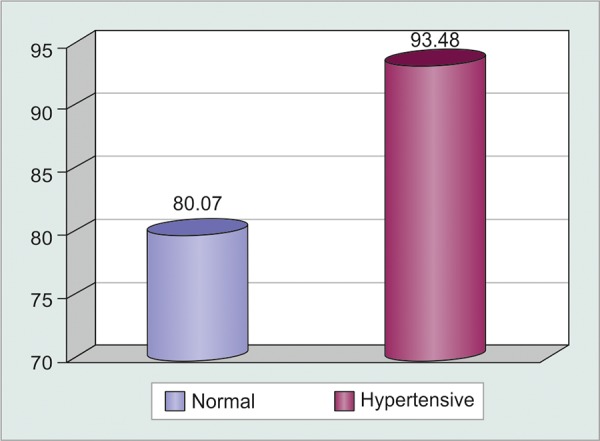
Comparison of the OPP of the normal and systemic hypertensive patients in mm Hg (p = 0)

The mean age of the patients was 57.5 years (36 to 78), and 68.5% were women (Table 1). There was a statistically significant difference in the OPP of the normal and systemic hypertensive patients (p < 0.05) (Graph 1). The difference of the OPP between these groups varied between 8.84 and 17.9 mm Hg (Table 2).

## DISCUSSION

Previous studies consider the normal IOP to be between 10 and 20 mm Hg.^10,11^ In this study, the median IOP was 14.5 mm Hg (SD 3.5), with 14.1 mm Hg (SD 3.8) in the normal and 14.7 mm Hg (SD 3.18) in the systemic hypertensive patients with no statistically significant difference. The MAP in normal patients was 94.2 mm Hg (SD 11.2) while that of the systemic hypertensive patients was 108.4 mm Hg (SD 14.78). This statistically significant difference (p = 0.000) confirms the good division of the groups. Leske et al^5^ investigated the relationship between the OPP and the incidence of open angle glaucoma (OAG) and found a relative risk of 3.1 for patients with OPP < 41.0 mm Hg. Fuchsjager-Mayrl et al^11^ concluded that the hemodynamics of ocular parameters are lower in patients with OAG in comparison with normal patients. Sehi et al^12^ described that the diurnal changes of the IOP were similar in normal and primary OAG patients; however, the OPP was significantly different between these groups with significant reductions at 7:00 am and after lunch, times when the IOP was in its maximum value. Our results demonstrated an OPP of 80.1 mm Hg (SD 10.6) in the normal patients and 93.5 mm Hg (SD 14.6) in the systemic hypertensive patients. The difference was statistically significant (p = 0) and confirmed a higher OPP in the systemic hypertensive patients.

**Table 1:** **Table 1:** Variables of the study

*Variables*		*Groups*		*n*		*Media*		*Median*		*SD*		*P25*		*P75*		*p-value*	
Age		Normal		47		54.1		53		8.9		49		61			
		Hypertensive		74		59.7		60.5		8.74		53		65		0.001*	
IOP		Normal		47		14.1		14.0		3.88		11		16			
		Hypertensive		74		14.7		14.7		3.18		12		16.6		0.345	
Systolic pressure		Normal		47		121.9		120		17.5		110		130			
		Hypertensive		74		143.6		140		20.5		130		160		0.000*	
Diastolic pressure		Normal		47		80.1		80		9.96		70		90			
		Hypertensive		74		90.7		90		13.46		80		100		0.000*	
MAP		Normal		47		94.2		93.3		11.2		83.3		103.3			
		Hypertensive		74		108.4		106.6		14.78		98.4		117.3		0.000*	
OPP		Normal		47		80.1		79.5		10.6		71.6		88.1			
		Hypertensive		74		93.5		92.9		14.6		84.7		102.7		0.000*	

IOP: Intraocular pressure; MAP: Median arterial pressure; OPP: Ocular perfusion pressure; SD: Standard deviation; *Statistically significant

**Table 2:** **Table 2:** Descriptive statistic of the POBF tonograph variability

*Variables*		*Groups*		*n*		*Media*		*Median*		*SD*		*P25*		*P75*		*p-value*	
IOP		Normal		39		15.4		16		3.62		12.9		17.7			
		Hypertensive		57		15.3		15.2		4.52		11.6		17.8		0.831	
AP		Normal		34		3.6		3.4		1.25		2.9		4.3			
		Hypertensive		50		3.9		3.7		1.92		2.75		4.5		0.498	
PV		Normal		34		7.4		7.2		2.54		5.6		8.4			
		Hypertensive		49		7.4		6.9		2.5		5.9		8.8		0.994	
PR		Normal		34		77.3		75.5		12.7		69.75		84.25			
		Hypertensive		49		79.6		76		16.11		68.5		88.5		0.505	
POBF		Normal		34		20.3		19.75		5.71		17.0		22.4			
		Hypertensive		49		20.1		20.1		5.28		16.85		23.25		0.895	

AP: Arterial pressure; IOP: Intraocular pressure; POBF: Pulsatile ocular blood flow tonograph; PR: Pulse rate; PV: Pulse volume; SD: Standard deviation

There is evidence of an abnormal association between ocular perfusion parameters and systemic blood pressure in patients with glaucoma. However, these data do refer to long-term adaptations of perfusion to the eye with blood pressure rather than a short-term increase in blood pressure.^13^

Despite this, we cannot quantify with any current diagnostic method, the real amount of blood in the anterior portion of the optic nerve or the level of existing gaseous exchange in this place. It is important to highlight that although was found that the systemic arterial hypertensive patients had a better ocular OPP in comparison to the normal population, they not necessarily have a better ocular nutrition. Usually in these chronic systemic arterial hypertensive patients coexist some microvascular changes, as the thickening of the intima vascular layer.

Yang et al,^14^ in the first study of its kind with a large sample size (n = 163), indicated normal average POBF of 11.16 ul/s for men and 14.03 ul/s for women, a statistically significant difference influenced by the greater cardiac frequency in women. In another study with a larger normal population (n = 664), Massey and Crowhurst obtained average POBF of 13.46 ul/s. Women had greater POBF (p = 0.001). Myopia, increase of the IOP, and older patients are related to reduced POBF value.^15^ Our study showed POBF values higher than described in the literature, 20.31 ul/s (SD 0.98) in normal patients and 20.15 ul/s (SD 0.75) in systemic hypertensive patients. This difference was not statistically significant (p = 0.895). None of the other parameters measured for the OBF were statistically significant.

The great variability of the measure of the POBF has been attributed to its relation with a great number of variables, including age, sex, cardiac frequency, pregnancy, corporal position, and ocular axial diameter.^2^ In this study, in many patients, the device was not able to take five complete cycles for the POBF calculation. While we observed a statistically significant difference in systemic AP among normal and hypertensive patients, a difference in the POBF between the groups was not seen, suggesting the existence of intrinsic factors related to the ocular hemodynamics or extrinsic factors related to the device or its software that regulates the OBF or makes its measuring difficult.

The results of this study suggest that although systemic hypertensive patients have a higher OPP in comparison to normal patients, this increase does not imply that they also have a higher OBF (as measured by the POBF tonograph). The higher OPP may be caused by chronic changes in the vascular net by the hemodynamics.

## CONCLUSION

There is a statistically significant difference in the OPP between normal and systemic hypertensive patients. More studies are required to evaluate the role of the OPP in ocular pathologies, especially in normal tension glaucoma. The results of this study suggest that although the systemic hypertensive patients have a higher OPP in comparison to a normal patient, this increase does not mean that they also have a higher OBF (as measured by POBF tonograph). This may be caused by chronic changes in the vascular net and in the blood hemodynamics in patients with systemic hypertension.
